# Evolution of the *SPX* gene family in plants and its role in the response mechanism to phosphorus stress

**DOI:** 10.1098/rsob.170231

**Published:** 2018-01-03

**Authors:** Na Liu, Wenyan Shang, Chuang Li, Lihua Jia, Xin Wang, Guozhen Xing, WenMing Zheng

**Affiliations:** State Key Laboratory of Wheat and Maize Crop Science, Collaborative Innovation Center of Henan Grain Crops, College of Life Sciences, Henan Agricultural University, Zhengzhou 450002, People's Republic of China

**Keywords:** plant, *SPX* gene, gene family, P signalling and homeostasis, functional analysis

## Abstract

Molecular and genomic studies have shown the presence of a large number of *SPX* gene family members in plants, some of which have been proved to act in P signalling and homeostasis. In this study, the molecular and evolutionary characteristics of the *SPX* gene family in plants were comprehensively analysed, and the mechanisms underlying the function of *SPX* genes in P signalling and homeostasis in the model plant species *Arabidopsis* (*Arabidopsis thaliana*) and rice (*Oryza sativa*), and in important crops, including wheat (*Triticum aestivum*), soya beans (*Glycine max*) and rapeseed (*Brassica napus*), were described. Emerging findings on the involvement of *SPX* genes in other important processes (i.e. disease resistance, iron deficiency response, low oxygen response and phytochrome-mediated light signalling) were also highlighted. The available data suggest that *SPX* genes are important regulators in the P signalling network, and may be valuable targets for enhancing crop tolerance to low P stress. Further studies on SPX proteins should include more diverse members, which may reveal SPX proteins as important regulatory hubs for multiple processes including P signalling and homeostasis in plants.

## 1. Introduction

The *SPX* family was named after *SYG1*, *PHO81* and *Xpr1*, the first three *SPX* gene members identified [1]. *SYG1* and *PHO81* encode yeast *gpa1* suppressor and cyclin-dependent kinase, respectively, while *Xpr1* codes for the xenotropic and polytropic retrovirus receptor 1 in humans. More and more studies have shown that *SPX* genes are involved in phosphorus (P) signalling and homeostasis and are prevalent in plants, and phosphate transport is impaired if the SPX domain is mutated [2]. P is an indispensable macroelement required for normal plant growth and development, and P content is quite high in plant tissues. P is not only an important component of membranes and nucleic acids, but also plays important roles in diverse physiological processes, including photosynthesis, enzyme activity regulation, respiration, signal transduction, oxidation–reduction reactions, energy metabolism and carbon metabolism [3–6]. Plants absorb P mainly from the soil through their roots [7,8]. Soil P exists primarily in the forms of calcium, iron and aluminium salts, and organic molecules, which are difficult for the roots to absorb [9]. This decreases the bioavailability of P, leading to an available P content in the soil that is far lower than that required for normal plant growth [10–12]. In plant cells, P concentration in the cytosol is approximately 60–80 µM [13], which is much higher than the concentration of available P in the soil (less than 10 µM) [14]. Thus, plants are usually under low P conditions. The phenotypic symptoms of P deficiency are mainly dark-green leaf colour, reduced elongation rate of shoot and decreased leaf size [15]. To improve crop yield in agricultural production, a large quantity of P fertilizers is often applied to solve the problem of P deficiency. However, this approach can cause not only water eutrophication but also overexploitation and consumption of phosphate ore, which is non-renewable. Solving this problem is critical to environmental protection and sustainable development. To adapt to P-deficient environments, plants undergo phenotypic changes in the root system to increase the absorption of P from the soil [16–19]. A series of P transport mechanisms at the molecular level are gradually established to overcome P starvation and to maintain P homeostasis, in which phosphate transporter is the basic effector involved in P uptake, transfer and storage [20,21]. These mechanisms are controlled by complex and sophisticated molecular regulatory networks. Recently, many genes have been identified and functionally linked to these molecular regulatory networks, including various protein-encoding genes and non-coding RNA genes [22]. Hence, the discovery of genes conferring low P tolerance has great importance.

In this study, the structural and evolutionary characteristics of the *SPX* gene family in plants were systematically analysed, the latest research progress on *SPX* gene functions was summarized and the mode of action of these genes in the P regulatory signalling network was discussed.

## 2. Structural characteristics of *SPX* genes and proteins in plants and their relationships with phosphorus metabolism

**Figure 1.**
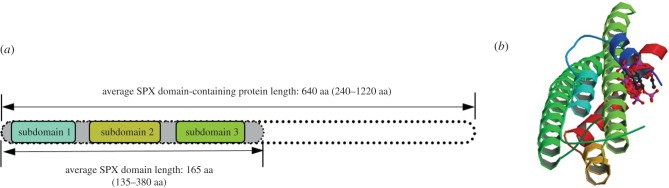
Main structural features of SPX proteins. (*a*) The N-terminal SPX domain can be divided into three well-conserved subdomains separated by low similarity regions, with 30–40 amino acids in each subdomain (Secco *et al*. [24]). (*b*) Crystal structure of the SPX domain in the Vtc4 protein of *Chaetomium thermophilum* in complex with inositol hexakisphosphate (InsP6). Classification: inositol phosphate-binding protein. SPX helical bundles provide a positively charged ligand-binding surface (Wild *et al*. [2]; http://www.rcsb.org/pdb/explore.do?structureId=5IJP).

The SPX domain found in eukaryotic proteins is rather conserved and has hydrophilic properties [23]. It is often located at the N-terminus of eukaryotic proteins [23]. SPX domain has an average length of 165 amino acids and can be divided into three subdomains with 30–40 amino acids in each (figure 1). They are separated from each other by low similarity regions [24]. In plants, SPX domains can be grouped into several distinct subfamilies: SPX proteins carrying only SPX domain, SPX–EXS proteins containing SPX and an EXS (ERD1, XPR1 and SYG1) domain, SPX–MFS proteins with SPX and the major facility superfamily (MFS) domain and SPX–RING proteins containing SPX and the RING-type zinc finger domain [25]. Many studies have shown that the SPX domain is closely related to P signalling and plays an important role in maintaining P homeostasis [22,26–28].

The SPX domain can indicate the phosphate status in fungal, plant and human cells. SPX domain-containing proteins are indispensable for the absorption, transport, storage and signal transduction of inorganic P in eukaryotes. Wild *et al*. [2] studied the ligand of the SPX domain and suggested that the domain provided a binding surface for small molecules (inositol polyphosphate signalling molecules, InsPs). In this way, the balance of P in plant cells can be regulated by the binding of different InsPs to SPX. In phosphate-deficient plant cells, InsPs bind to SPX domains, being able to interact with several other proteins involved in the regulation of P signalling in plants [2,29]. If the SPX domain is mutated, then phosphate transport capacity is impaired [2], highlighting the unique importance of the SPX domain in P metabolism.

P exists in different molecular forms in plants to serve their needs at different times [30]. One form is inorganic polyphosphate (polyP). PolyP includes hundreds of types of phosphoric anhydrides, which can be hydrolysed to meet the needs of various molecular processes [31–33]. In yeast, the vacuolar transporter chaperone (VTC) complex can synthesize polyP [34]. The VTC is a fairly large protein complex (Vtc1–5) located on the vacuole membrane [35,36]. Approximately 80% of VTC proteins contain an SPX domain, which may contribute to P homeostasis in the cells [5]. Additionally, an important role for the phosphate starvation response 1 (PHR1) protein has been identified in the P signalling network. This MYB-like transcription factor is homologous to phosphorus starvation response 1 (PSR1), which participates in the P sensing process in *Chlamydomonas reinhardtii* [37,38]*.* PHR1 regulates the expression of *AtACP5*, *AtIPS1*, *PHT1.1* and *RNS1* [39,40], as well as the expression of several PSR genes, including *microRNA399* and the *SPX* genes [38], by binding to their promoters through the *cis*-element PHR1-binding sequence (P1BS; GNATATNC) [40–42].

## 3. Evolutionary analysis of *SPX* gene family in plants

**Figure 2.**
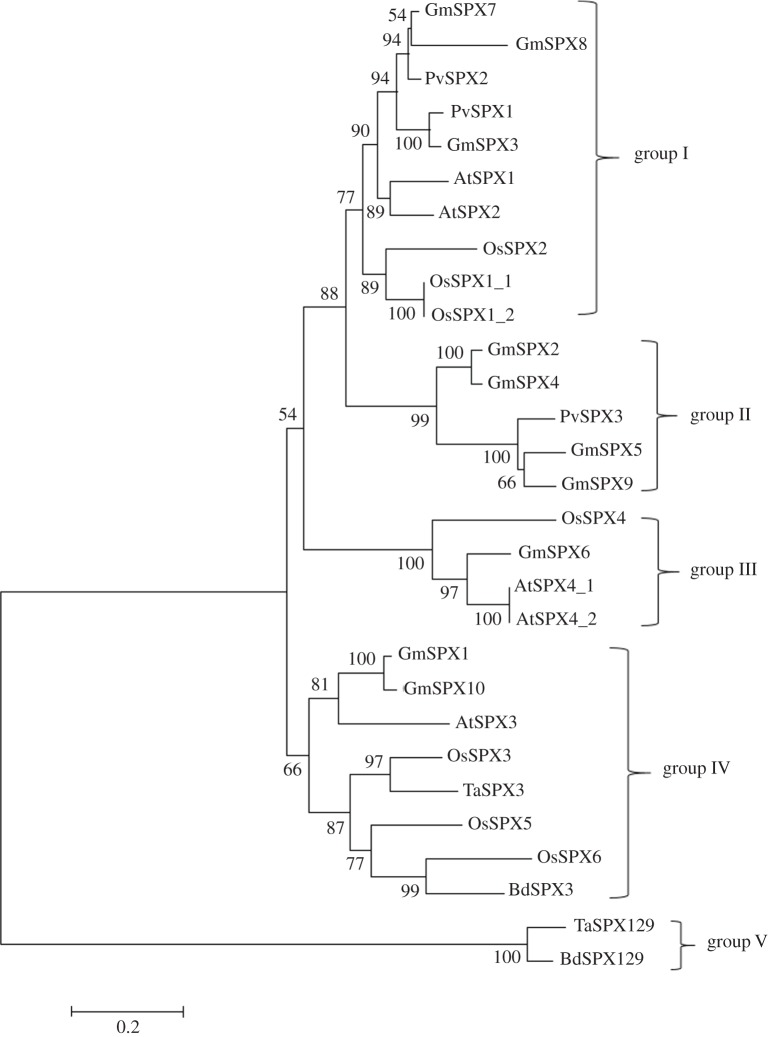
Phylogenetic tree of SPX domain-containing proteins from different plant species. The tree was constructed with the neighbour-joining method by MEGA with 1000 bootstrap replicates. At, *Arabidopsis thaliana*; Os, *Oryza sativa*; Pv, *Phaseolus vulgaris*; Gm, *Glycine max*; Ta, *Triticum aestivum*; Bd, *Brachypodium distachyon*.

Through analysis of existing plant genomic sequences, 20 *SPX* gene family members have been identified in *Arabidopsis*, including four *SPX* genes whose deduced proteins contain only the SPX domain [26]. Meanwhile, 15 *SPX* gene family members are identified in rice, including six *SPX* genes whose products carry only the SPX domain [24]. Numerous *SPX* gene family members also exist in the genomes of legumes and other important crops [43,44]. A phylogenetic tree was constructed for some of the important *SPX* genes using the multi-sequence alignment generated by ClustalW and the neighbour-joining method with 1000 bootstrap replicates in MEGA software (figure 2). The results showed evolutionary divergence in *SPX* genes, and the compared genes were clustered into five types of sub-structures (numbered I, II, III, IV and V). Most of the *SPX* genes fell into types I and IV, whereas no more than five genes fell into each of the other three types. *GmSPX8*, *GmSPX5*, *OsSPX4*, *OsSPX6* and *TaSPX129* genes exhibited the most rapid evolution for each type. The paralogues of these genes included *AtSPX1*/*AtSPX2*, *GmSPX2*/*GmSPX4*, *GmSPX5*/*GmSPX9*, *OsSPX1*/*OsSPX2*, *GmSPX1*/*GmSPX10* and *OsSPX5*/*OsSPX6*. Each gene may evolve under different evolutionary pressure and may possibly acquire new function during the course of evolution.

**Figure 3.**
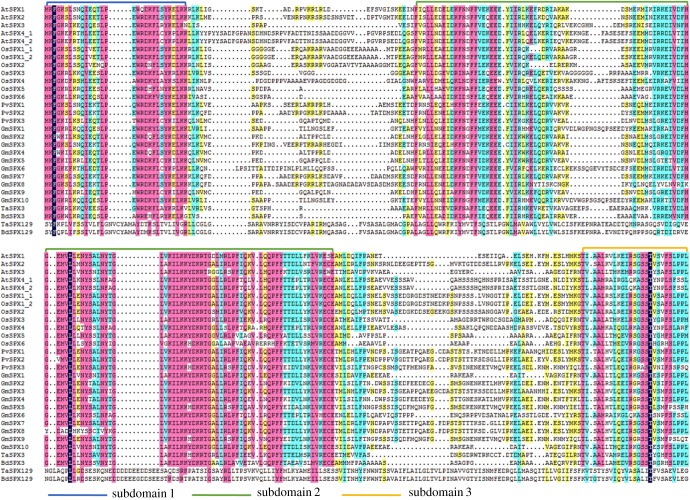
Multiple alignment of the SPX domains in different SPX proteins. The multiple alignment was generated using DNAMAN with different colours representing different homology of amino acids. The three subdomains are distinguished by coloured brackets. At, *Arabidopsis thaliana*; Os, *Oryza sativa*; Pv, *Phaseolus vulgaris*; Gm, *Glycine max*; Ta, *Triticum aestivum*; Bd, *Brachypodium distachyon*.

A multiple sequence alignment of the SPX domains was analysed (figure 3). The result showed a high degree of similarity in the SPX domains in *Arabidopsis*, rice, common beans, soya beans, wheat and *Brachypodium*. The SPX domains in TaSPX129 and BdSPX129 were of SPX–MFS type, and have an increased length, whereas the remaining SPX domains ranged from 200 to 400 amino acids. Amino acid point mutations are the main sources of variation, which may affect the role of *SPX* genes in P regulatory networks in plants. Notably, the SPX domains in TaSPX129 and BdSPX129 had many insertions (figure 3); whether this is caused by the presence of an additional MFS domain is a matter for further study.

## 4. Research progress on the functional analysis of *SPX* genes in plants

A large number of SPX domain-containing proteins have been identified in plants [11]. Here, SPX proteins refer to the proteins that contain only the SPX domain. Owing to their presence in different subcellular structures, they may have different functions in the P signalling network. Here, we summarize the studies on the genes whose proteins contain only the SPX domain, including *SPX1–4* in *Arabidopsis*, *SPX1–6* in rice, *SPX1–10* in soya beans, *SPX1–3* in common bean and *TaSPX129* in wheat (table 1).

**Table 1.** List of the plant *SPX* genes whose function has been analysed to some extent. N, cell nucleus; M, cell membrane; C, cytoplasm; +, increase; +(*), increase (except seeds); +(**), increase (except flowers and seeds); =, no difference; −, decrease; Pr, positive regulation; Pr*, positive regulation (except *PvPDR2-like*); Nr, negative regulation.

species	gene	protein location	expression after Pi starvation	regulation of PSI gene	main functional characteristics	source
*Oryza sativa*	*OsSPX1*	N	+	Nr	OsSPX1 can interact with OsPHR2 and acts as a negative regulator of OsPHR2. OsSPX1 regulates *OsSPX2*, *3* and *5* at the transcriptional level, and the repression of *OsSPX1* results in excessive P accumulation in the shoot	Wang *et al.* [27,28]
	*OsSPX2*	N	+		OsSPX2 can interact with OsPHR2 and acts as a negative regulator of OsPHR2. PHR2, SPX1 and SPX2 constitute a regulatory feedback loop in P signalling	Wang *et al*. [28,45]
	*OsSPX3*	N/C	+	Nr	OsSPX3 plays an important role in OsIPS1/miR399-mediated long distance regulation on OsPHO2 and acts as a negative regulator of OsPHR2. OsSPX3 negatively regulates the root-to-shoot transportation of P. Overexpression of *OsSPX3* inhibits plant growth, which is more severe under P-deficient conditions	Wang *et al.* [28] Shi *et al*. [46]
	*OsSPX4*	N/C	=		OsSPX4 can interact with OsPHR2 in the cytoplasm and inhibits translocation of PHR2 into the nucleus. OsSPX4 functions as a negative regulator of PHR2 and can affect the activity of OsPHR2, sequentially regulating downstream gene expression	Lv *et al*. [47]
	*OsSPX5*	N/C	+	Nr	*OsSPX5* and *OsSPX3* are paralogous genes. SPX3/5 proteins act as repressors of PHR2. Overexpression of *SPX3* and *SPX5* completely rescues the excessive shoot of P accumulation. SPX3/5 negatively regulates P transport from roots to leaves with redundant function	Shi *et al*. [46] Zhang *et al*. [43]
	*OsSPX6*		+		*OsSPX6*, as a paralogue of *SPX3/5*, may play a compensatory role	Shi *et al*. [46]
*Arabidopsis thaliana*	*AtSPX1*	N	+	Pr	AtSPX1 can interact with AtPHR1 and may act as a negative regulator of AtPHR1 in P concentration	Duan *et al*. [26] Qi *et al*. [48]
	*AtSPX2*	N	+		AtSPX2 can interact with AtPHR1 in the cell nucleus. AtSPX1 and AtSPX2 have functional redundancy with one another	Puga *et al*. [49]
	*AtSPX3*	M/C	+	Nr	Partial repression of *AtSPX3* can exacerbate phosphate-deficiency symptoms, alter P allocation and enhance the expression of a subset of phosphate starvation responsive genes including *AtSPX1*	Duan *et al*. [26]
	*AtSPX4*	M	−		AtSPX4 can interact with AtPHR1 in the cytoplasm	Duan *et al*. [26]
*Glycine max*	*GmSPX1*	N/C	+(*)	Nr	GmSPX1 interacts with a newly identified P starvation-induced transcription factor GmMYB48, and this interaction may represent a potential suppressor of P signalling network in soya bean	Zhang *et al*. [43]
	*GmSPX2*, *4*, *6*, *9* and *10*	N/C	+		—	Yao *et al*. [50]
	*GmSPX3*, *7* and *8*	N	+		*GmSPX3* overexpression results in increased P concentration in both leaf and root tissues under high P conditions, which correlates with elevated transcript levels of several PSI genes in the root hairs	Yao *et al*. [50]
	*GmSPX5*	N/C	+(**)		—	Yao *et al*. [50]
*Phaseolus vulgaris*	*PvSPX1*	N	+	Pr*	Overexpression of *PvSPX1* results in increased P concentration in the roots, morphological change in root hairs, inhibition of main root growth, more numerous lateral roots and upregulated transcription of 10 PSR genes	Yao *et al*. [44]
	*PvSPX2*	N	+	Pr*	PvSPX2 participates in P signalling pathway in both shoot and root tissues. Overexpression of *PvSPX2* results in increased transcription of several genes downstream from *PvSPX1*, suggesting that PvSPX2 might have a similar regulatory role as PvSPX1	Yao *et al*. [44]
	*PvSPX3*	N/C	+	=	PvSPX2 participates in P signalling pathway in both shoot and root tissues. *PvSPX3* expression is less sensitive to P deficiency compared with that of *PvSPX1* and *PvSPX2*	Yao *et al*. [44]
*Triticum aestivum*	*TaSPX129*		+		—	Fang *et al*. [51]
	*TaSPX*				TaSPX participates in high temperature-induced resistance to wheat stripe rust	Wei *et al*. [52]

### 4.1. Functional analysis of *SPX* genes in *Arabidopsis*

**Figure 4.**
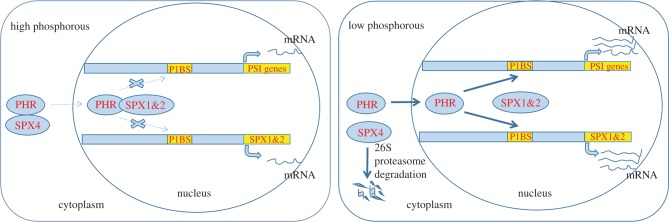
Interaction of SPX1, SPX2 and SPX4 with PHR under high or low P conditions. Under high P conditions, SPX1 and SPX2 in the nucleus and SPX4 in the cytoplasm bind PHR with high affinity, thus inhibiting PHR binding to the P1BS motif in the promoters of PSI genes and leading to repression of the transcription of PSI genes, including *SPX1* and *SPX2*. Under low P conditions, SPX4 in the cytoplasm is degraded via the 26S proteasome pathway, which promotes the targeting of PHR to the nucleus, thereby releasing PHR to activate downstream PSI gene expression. Meanwhile, SPX1 and SPX2 in the nucleus interact with PHR at a low affinity, which facilitates PHR to bind to the P1BS motif in the promoters of PSI genes, further enhancing the expression of PSI genes, including *SPX1* and *SPX2*. The thick arrow denotes enhancement. The dotted lines represent reduced effects. This working model is drawn based on the studies of *SPX1*, *SPX2* and *SPX4* genes in *Arabidopsis* and rice (Lv *et al*. [47]; Wang *et al*. [27,28]; Zhou *et al*. [53]; Puga *et al*. [49]).

Twenty SPX domain-containing proteins were found in *Arabidopsis*, among which four proteins contained only the SPX domain (AtSPX1–AtSPX4) [26]. AtSPX1, localized in the nucleus, is a P-dependent suppressor of *AtPHR1* in *Arabidopsis* [53]. *AtPHR1* overexpression results in an increase in P concentration in the shoot and induces the expression of a series of Pi starvation-induced (PSI) genes that encode phosphate transporter, phosphatase or RNase [54,55]. Co-immunoprecipitation experiment showed that the AtSPX1/AtPHR1 interaction was strongly dependent on P level. AtSPX1 is a competitive suppressor that binds AtPHR1 through its recognition sequence. The working model in figure 4 depicts the interactions of AtSPX with AtPHR1 in response to cellular Pi concentration for PSI transcription. Under high P conditions, AtSPX1 has a high binding affinity for AtPHR1, and thus the process by which AtPHR1 regulates PSI genes through the P1BS is inhibited, resulting in a decrease in PSR gene expression. Under P deficiency conditions, the AtSPX1/AtPHR1 interaction weakens, thus facilitating the binding of AtPHR1 to the P1BS to regulate PSR gene expression [49].

In *Arabidopsis*, no significant phenotypic differences have been found among the single-gene knockout mutants of *Atspx1*, *Atspx2* and *Atspx4* under P-sufficient or -starvation conditions. However, in plants with *AtSPX1* overexpression, the expression levels of some PSI genes (i.e. *ACP5*, *PAP2* and *RNS1*) are significantly increased regardless of P concentration, thus suggesting that *AtSPX1* may function in the transcriptional regulation of P starvation. Additionally, inhibition of *AtSPX3* through RNAi can change the phenotypes and gene expression levels under P-starvation conditions, rendering an increase in P concentration in the shoot tissues and a reduced P concentration in the roots [26,43]. The expression levels of *AtPHT1–4*, *AtPHT1–5*, *AtACP5*, *AtRNS* and *AtAT4* in *spx3* deletion mutants are increased irrespective of P concentration [43], indicating that AtSPX3 is a negative regulator of the signalling process of P starvation. Collectively, these results indicate that SPX proteins have functional redundancy with one another and can serve as an important role in regulating Pi signalling and homeostasis in plants.

### 4.2. Functional analysis of *SPX* genes in rice

P is an important nutrient element that limits the yield of rice. Studies of P relevant genes in rice, especially *SPX* genes, can potentially aid rice yield improvement. A total of 15 SPX domain-containing proteins have been identified in rice, of which only six are SPX proteins (OsSPX1–OsSPX6) [5]. OsSPX1 inhibits P uptake and P-starvation signalling through negative feedback regulation [27,55]. OsSPX1 is induced by P starvation in the roots, and inhibition of *OsSPX1* by RNAi leads to an excessive accumulation of P and thus induces severe toxicity. This phenotype is similar to that observed in the plants overexpressing *OsPHR2* and the *pho2* mutant. *OsPHR2* overexpression leads to increased PSI gene expression, including *IPS1* and *PT2*, which promotes excessive P absorption and accumulation and results in leaf necrosis. Quantitative polymerase chain reaction (qRT-PCR) assay showed that *OsSPX1* expression was strongly induced in the plants with *OsPHR2* overexpression and the *pho2* mutant, suggesting that *OsSPX1* may function downstream from *PHO2* and *OsPHR2*. Wang *et al.* [27] analysed the expression levels of 10 genes involved in the rice P-starvation signalling pathway. *OsPT2* and *OsPT8* were significantly induced in *OsSPX1* RNAi plants, pointing to increased P transport and accumulation. By contrast, *OsSPX1* overexpression inhibited the expression of 10 phosphate starvation-mediated genes, including *IPS1*, *IPS2*, *OsPAP1*, *OsSQD2* (*sulfo quinovosyl diacylglycerol 2*), *miR399d*, *miR399j*, *OsPT2*, *OsPT3*, *OsPT6* and *OsPT8*. However, in the double mutant plants with overexpression of *OsPHR2* and *OsSPX1*, P concentration and PSR gene expression levels were basically the same as those in wild-type plants, which indicated that OsSPX1 was a negative regulator of OsPHR2-mediated signal transduction. Previous studies found that overexpression of *OsSPX1*, *OsSPX2*, *OsSPX3*, *OsSPX4* and *OsSPX5* attenuated the phenotype of *OsPHR2* overexpression [45–47,53]. Thus, OsSPX1, OsSPX2 and OsSPX4 may interact with OsPHR2 and inhibit its binding to the P1BS *cis*-acting element (figure 4). The interaction between SPX proteins and PHR1/2 was strongly dependent on P concentration [45,47]. Knockout of *OsSPX1*, *OsSPX2* and *OsSPX4* results in P accumulation in the shoot and significant leaf tip necrosis [45,47]. P accumulation and leaf necrosis also occurred in the *Osspx3* and *Osspx5* double mutant, and the expression levels of PSI genes, including *IPS1*, *miR399*, *PT2*, *miR827*, *PAP10* and *SQD2*, were significantly upregulated [46]. This observation indicated that *OsSPX3* and *OsSPX5* were homologous and that they responded to P stress at the transcriptional and post-transcriptional levels. Collectively, the data gathered so far support the function of the studied *OsSPX* genes in rice tolerance to P deficiency by regulating P acquisition and its transport from roots to leaves.

### 4.3. Functional analysis of *SPX* genes in legumes

Phylogenetic analysis demonstrated that GmSPXs 1–10 can be divided into three groups [43]. Quantitative PCR assay showed that the expression of these genes was significantly increased under low P conditions and decreased rapidly one day after P supplementation [43]. The expression of these genes was highly sensitive to low P conditions. Overexpression of *GmSPX3* led to increased P concentration in both leaf and root tissues, and increased transcriptional levels of seven PSI genes in the root hairs, under high P conditions [44]. Analysis of *GmSPX1* overexpression in *Arabidopsis spx3* mutant showed that GmSPX1 negatively regulated many PSR genes, including *AtPHT1–4*, *AtPHT1–5*, *AtACP5*, *AtRNS* and *AtAT4*, in a P level-dependent manner [43]. Furthermore, GmSPX1 interacted with a newly identified P starvation-induced transcription factor GmMYB48, and this interaction may represent a potential suppressor module of the P signalling network in soya bean [43]. Three SPX proteins (PvSPX1–PvSPX3) have been found in common bean (*Phaseolus vulgaris*), and their expression levels were significantly increased in roots and leaves under P-starvation conditions [44]. PvSPX1 is localized in the nucleus and exhibits a more sensitive and rapid response to P starvation. Overexpression of *PvSPX1* resulted in an increase in P concentration in root tissues, a configuration change of root hairs, growth inhibition of the main root and an increase in the number of lateral roots, accompanied by upregulated transcription of 10 PSR genes [44]. Further studies showed that *PvPHR1* overexpression increased *PvSPX1* transcription level, and thus *PvSPX1* may act downstream from *PvPHR1* [44,50].

### 4.4. Functional analysis of *SPX* genes in wheat

P deficiency is also a primary factor constraining the yield of wheat [15]. Therefore, identification of P-regulated genes and breeding of low P-tolerant cultivars are of prime importance to increasing global wheat productivity without excessive use of P fertilizers. Owing to the possession of a complex hexaploid genome, functional analysis of P-regulated genes (including *SPX* members) in common wheat (2*n* = 6*x* = 42) is lagging behind that in *Arabidopsis* and rice. Shang *et al.* [56] found that *TaSPX3* was strongly induced by low P stress, but became significantly downregulated when P supply was restored; the expression profile of *TaSPX3* differed among cultivars, indicating that the mechanism of low P stress response may vary among different wheat genotypes. Shukla *et al*. [57] demonstrated that the relative transcriptional level of *TaSPX1* was higher in the aleurone than in the endosperm in developing wheat grains, which paralleled the accumulation of more P in aleurone tissues.

### 4.5. Functional analysis of *SPX* genes in rapeseed

Du *et al*. [11] analysed 69 *SPX* gene family members in rapeseed (*Brassica napus*) and found that the expression levels of different *BnaSPX* genes differed under P-starvation conditions. The expression levels of nine genes in the *SPX* subfamilies were significantly induced by P starvation and rapidly declined upon P supplementation. Analysis of two *BnaSPX1* genes (i.e. *BnaA2.SPX1* and *BnaC3.SPX1*) in transgenic *Arabidopsis* revealed functional difference between them: the transgenic lines of *BnaA2.SPX1*, but not those of *BnaC3.SPX1*, showed retarded growth and higher sensitivity to P deficiency when compared with wild-type control [11]. In two other studies, *BnSPX3;1* and *BnSPX3;2* were found specifically induced by P deficiency and that the induction was rapid and reversible [58,59]. Unlike P deficiency, the deprivation of other nutrients (N, K, S or Fe) did not affect the transcription of *BnSPX3;1* and *BnSPX3;2*, and thus the two genes may be used as markers for assessing P-starvation status in plants [58,59].

## 5. Discussion and prospects

The available studies clearly suggest that SPX proteins occupy a very important position in the P signalling network, which is tightly related to P uptake, transport, storage and homeostasis. Most of the studied *SPX* genes are low P inducible and can influence the transcription of downstream PSI (PSR) genes by regulating PHR activity, likely via controlling the movement of PHR from the cytoplasm to the nucleus and by decreasing the binding of PHR to the P1BS *cis*-element [45,47,49]. Not surprisingly, current understandings on *SPX* genes are largely based on the data from model plants (i.e. *Arabidopsis* and rice). The results from complex crop plants (e.g. legumes, common wheat and rapeseed) are much less. Nevertheless, they complemented and expanded the insights obtained from model species, and yielded potential clues and targets for enhancing crop tolerance to low P stress. Considering the urgent need in developing P-efficient cultivars [60], more efforts should be devoted to studying *SPX* gene functions in crop plants. The accumulation of ever more genomic resources [61], as well as the rapid development of gene-editing technologies for diverse plant species [62], will facilitate such efforts.

Past investigations have mainly concerned relatively simple SPX proteins, i.e. those with only one SPX domain. Future studies should cover more complex SPX proteins that carry extra domains in addition to SPX. The presence of extra domains may confer multiple functions to SPX proteins. This possibility may be illustrated by the analysis of *Arabidopsis* PHO1 (AtPHO1), which carries the EXS domain in addition to SPX. Although originally found required for xylem loading of inorganic phosphate [63,64], recent investigations have indicated the likely involvement of AtPHO1 in the cross talk among P, sucrose and phytohormone signalling pathways [65].

Future studies will also shed new light on the involvement of SPX proteins in other vital plant processes. There is emerging evidence for the participation of SPX domain-containing proteins in disease resistance [52], iron deficiency response [66], low oxygen response [67] and phytochrome-mediated light signalling [68]. Considering the diverse and fundamental roles of P in cellular organisms, it may not be surprising to find that SPX proteins act as important regulatory hubs for multiple processes including the fine tuning of P signalling and homeostasis in plants.

## Data accessibility

This article has no additional data.
